# Supplementary material for: A pore-forming protein drives macropinocytosis to facilitate toad water maintaining
Source: Commun Biol. 2022 Jul 22;5:730. doi: 10.1038/s42003-022-03686-1 (PMC9307623; doi:10.1038/s42003-022-03686-1)
Supplement: Supplementary file 2 — Supplementary Information [file 42003_2022_3686_MOESM2_ESM.pdf]

Supplemental Information

Supplementary Figures and Table

Supplementary Figure 1. βγ-CAT is involved in responses to osmotic stress.

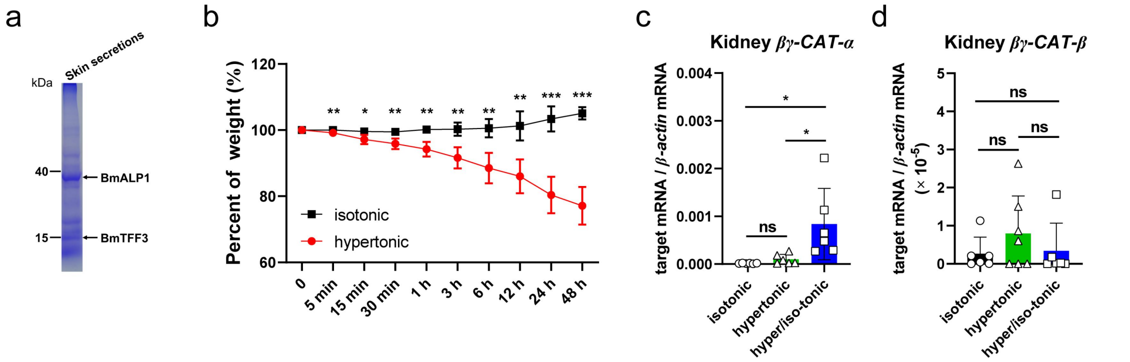

(**a**) 5 μg freeze-dried skin secretions were analyzed by SDS-PAGE with Coomassie blue staining. (**b**) Percentage change curves of toad weights in isotonic or hypertonic Ringer’s solution within 48 hours (*n* = 4). (**c, d**) The expression of *βγ-CAT* subunits in toad kidney as analyzed by real-time fluorescent quantitative PCR after toads were placed in isotonic, hypertonic and hypertonic/isotonic Ringer’s solution (transferring toads from hypertonic to isotonic Ringer's solution) for 3 hours (*n* = 6). All data of the weight change and gene expression represent the mean ± SD. ns ($P \geq 0.05$), *$P < 0.05$, **$P < 0.01$ and ***$P < 0.001$ by unpaired *t* test. All data are representative of at least two independent experiments.

Supplementary Figure 2. The endogenous secretion and cytotoxicity of βγ-CAT and toad *B. maxima* UB AQP analysis.

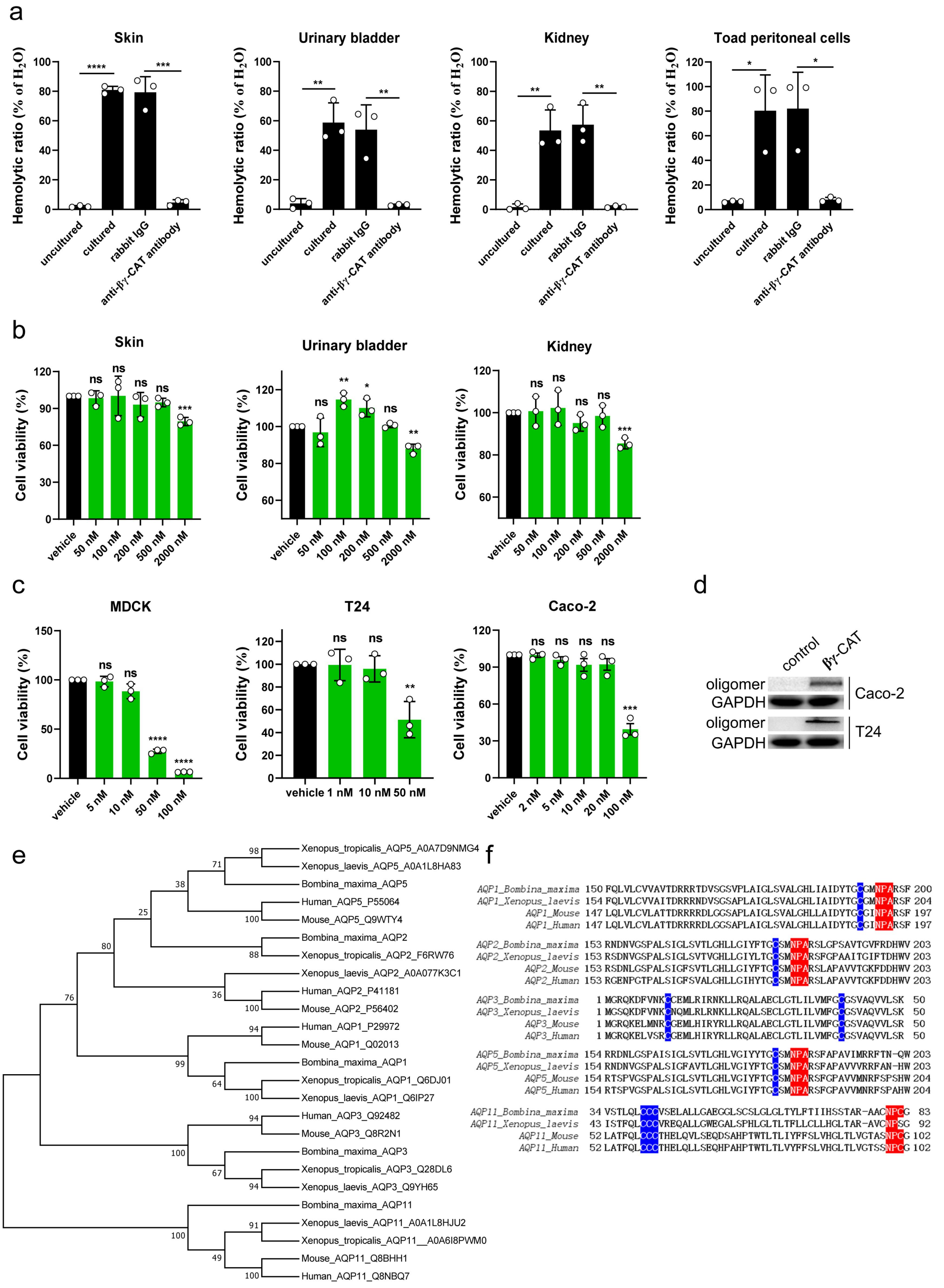

(**a**) The endogenous secretion of βγ-CAT was analyzed by its hemolytic activity on human erythrocytes. The samples assayed were uncultured

supernatant, cultured supernatant, and cultured supernatant containing 50 μg mL^{-1} rabbit IgG supernatant or 50 μg mL^{-1} anti-βγ-CAT antibody supernatant for toad skin, UB, kidney and peritoneal cells. (**b**) Cytotoxicity assays of βγ-CAT on digestive epithelial cells of toad skin, UB and kidney. The cells were treated by βγ-CAT for 3 hours, then the cell viability was determined by MTS. (**c**) Cytotoxicity assays of βγ-CAT on MDCK, Caco-2 and T24 cells as assayed in (b). (**d**) Caco-2 and T24 cells were treated with or without βγ-CAT for 15 minutes. The appearance of βγ-CAT oligomers in the treated cells was determined by Western blotting. (**e**) Molecular phylogenetic analysis of AQPs in toad *B. maxima* UB and other species by Maximum Likelihood method. (**f**) Sequence alignment of AQPs in toad *B. maxima* UB and other species by Clustal Omega. NPA/NPC (red) is the signature motif of AQPs, and cysteine (blue) is the mercury binding site. The results are reported as mean ± SD of triplicate samples in b, c. ns ($P \geq 0.05$), *$P < 0.05$, **$P < 0.01$, ***$P < 0.001$ and ****$P < 0.0001$ by unpaired *t* test. All data are representative of at least two independent experiments in a-c.

Supplementary Figure 3. βγ-CAT promotes macropinocytosis.

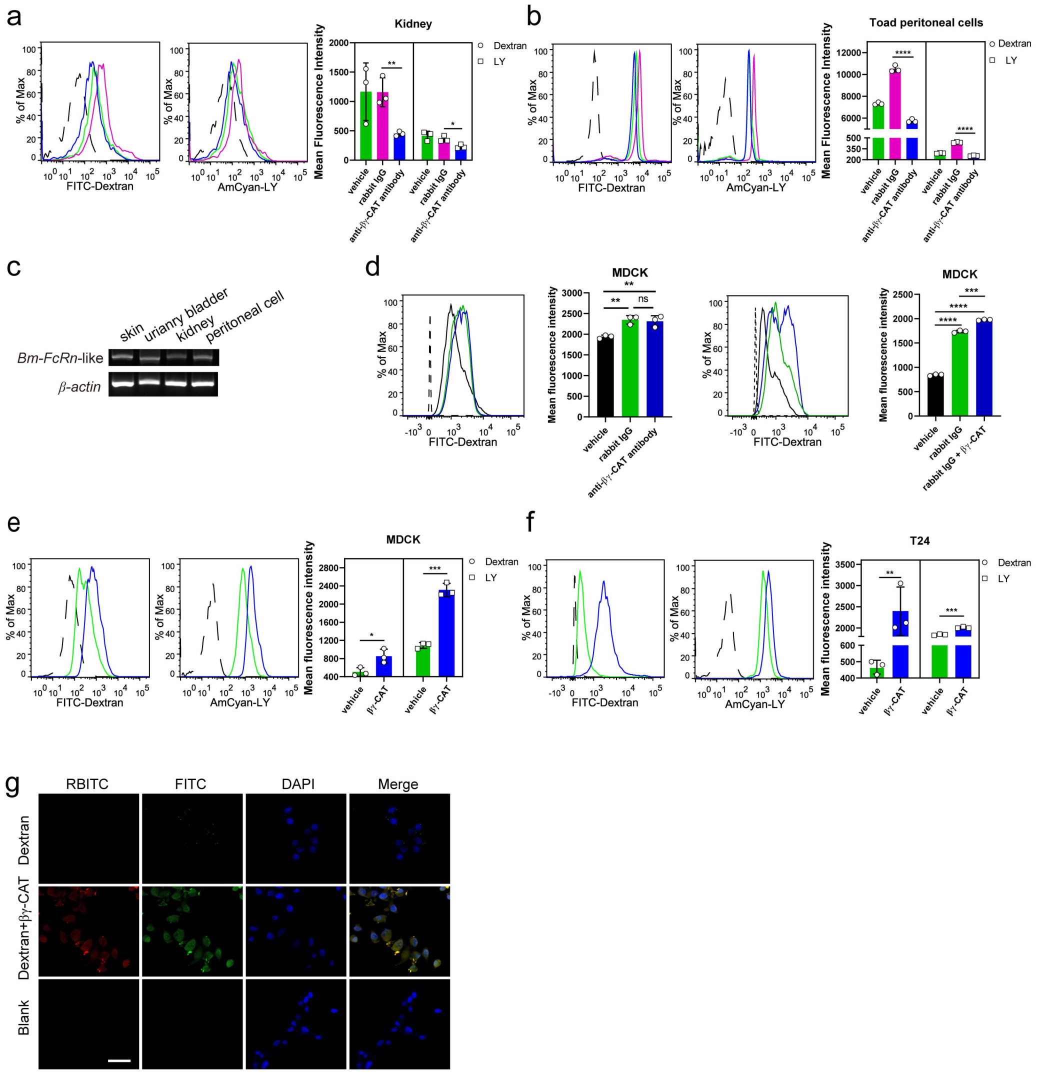

(**a, b**) Immunodepletion of endogenous βγ-CAT decreased macropinocytosis. The mean fluorescence intensity of 70 kDa FITC-label dextran and Lucifer Yellow (LY) in toad kidney epithelial cells (a) and peritoneal cells (b) was determined by flow cytometry. The cells were incubated with 50 μg mL^{-1} anti-βγ-CAT antibodies to immunodeplete endogenous βγ-CAT for 30 minutes, and rabbit IgG was used as an antibody control. Vehicle refers to an antibody absent control. Then the cells were incubated with 100 μg mL^{-1} of LY or FITC-label dextran at 37℃ for 30 minutes. The black dotted line refers to normal (blank control). (**c**) *FcRn*-like gene expression in toad cells. (**d**) Exogenous rabbit IgG enhanced the pinocytosis of MDCK cells. The mean fluorescence intensity of 70 kDa FITC-label dextran in MDCK cells was determined by flow

cytometry. The cells were incubated with 50 μg mL^{-1} rabbit IgG or anti-βγ-CAT antibodies for 30 minutes. Vehicle refers to an exogenous rabbit IgG absent control. Then the cells were incubated with 100 μg mL^{-1} of FITC-label dextran at 37℃ for 30 minutes with and without 10 nM βγ-CAT. The black dotted line refers to normal (blank control). (**e, f**) The mean fluorescence intensity of LY and FITC-label dextran in MDCK (e) and T24 (f) cells as determined by flow cytometry with or without additional 10 nM or 5 nM βγ-CAT, respectively. The black dotted line refers to normal (blank control). (**g**) Localization of RBITC-label βγ-CAT (red) and FITC-label dextran (green) in MDCK cells with or without the treatment with 10 nM RBITC-label βγ-CAT, respectively, for 15 minutes by immunofluorescence (Scale bars, 30 μm). The results are reported as mean ± SD of triplicate samples in a-d. *P < 0.05, **P < 0.01, ***P < 0.001 and ****P < 0.0001 by unpaired t test. All data are representative of at least two independent experiments.

Supplementary Figure 4. βγ-CAT enhances exosome release.

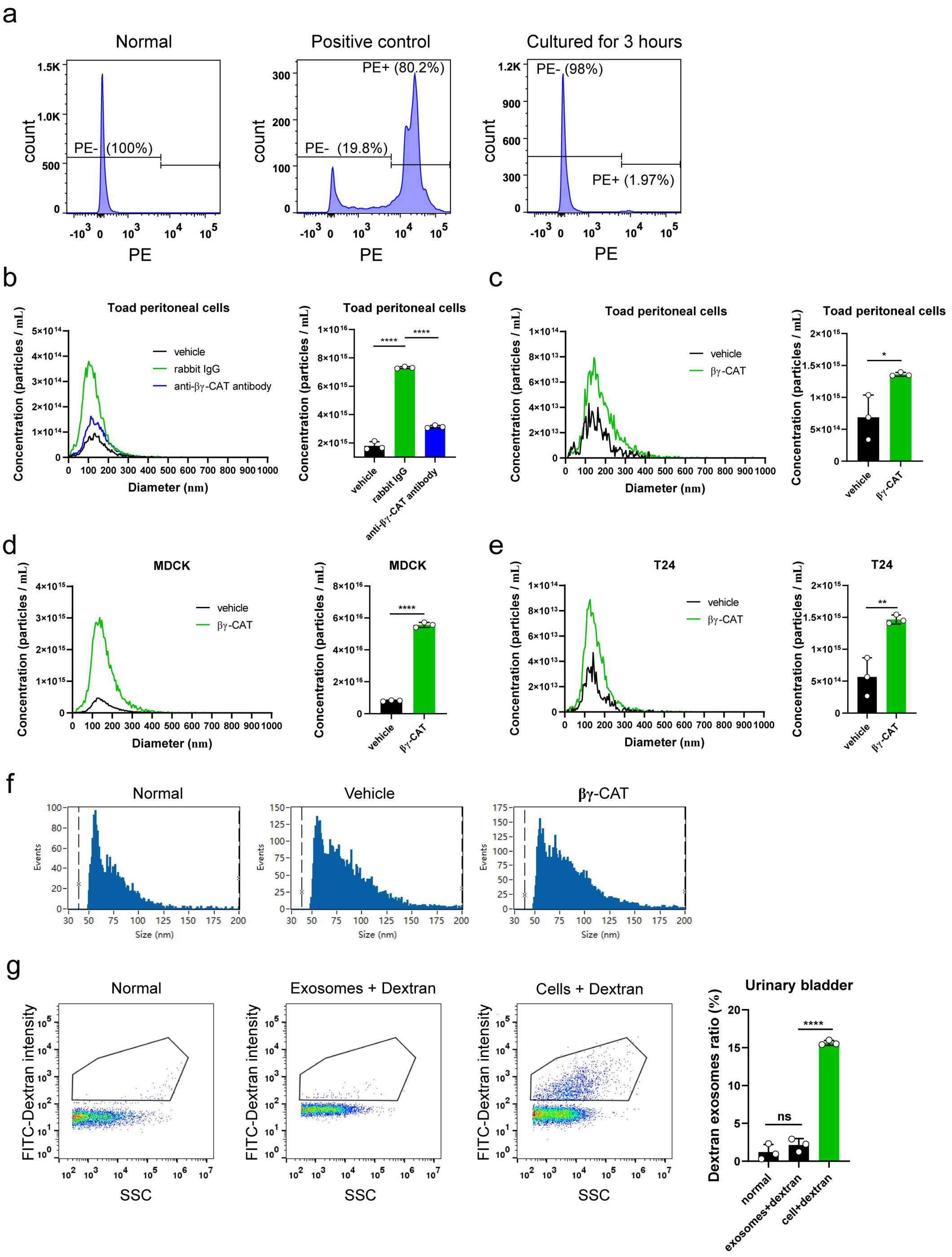

(**a**) The survival rate of toad UB epithelial cells cultured *in vitro* for 3 hours was analyzed by flow cytometry. Digested UB epithelial cells were cultured in isotonic Ringer's solution at room temperature for 3 hours and then the cells were incubated with 500 ng mL^{-1} propidium iodide (PI) PBS for 10 minutes.

The positive control group was treated with 4% paraformaldehyde for 10 minutes before PI staining. (**b, c**) NTA analysis of the diameter and particle concentration of exosomes isolated from toad peritoneal cells which were cultured in the presence or absence of 50 μg mL^{-1} anti-βγ-CAT antibodies (b) or 50 nM βγ-CAT (c) at room temperature for 3 hours. (**d, e**) NTA analysis of the diameter and particle concentration of exosomes isolated from MDCK (d) and T24 (e) cells cultured in the presence or absence of 10 nM (MDCK) or 5 nM (T24) βγ-CAT for 3 hours at 37℃, respectively. (**f**) Nanoflow cytometry analysis of the diameter of exosomes derived from toad UB epithelial cells cultured for 3 hours in the presence of 1mg mL^{-1} dextran with or without the addition of 50 nM βγ-CAT. (**g**) Comparison of dextran exosome percentage between exosomes cultured directly with 1 mg mL^{-1} dextran medium and those collected from UB epithelial cells cultured with 1mg mL^{-1} FITC-label dextran medium for 3 hours. The data were obtained by Nanoflow Cytometry and the quantitative result was presented as a bar chart. The results are reported as mean ± SD of triplicate samples in b-e, g. *$P < 0.05$, **$P < 0.01$ and ****$P < 0.0001$ by unpaired *t* test. All data are representative of at least two independent experiments.

92 **Supplementary Figure 5. Uncropped and unedited blot/gel images.**

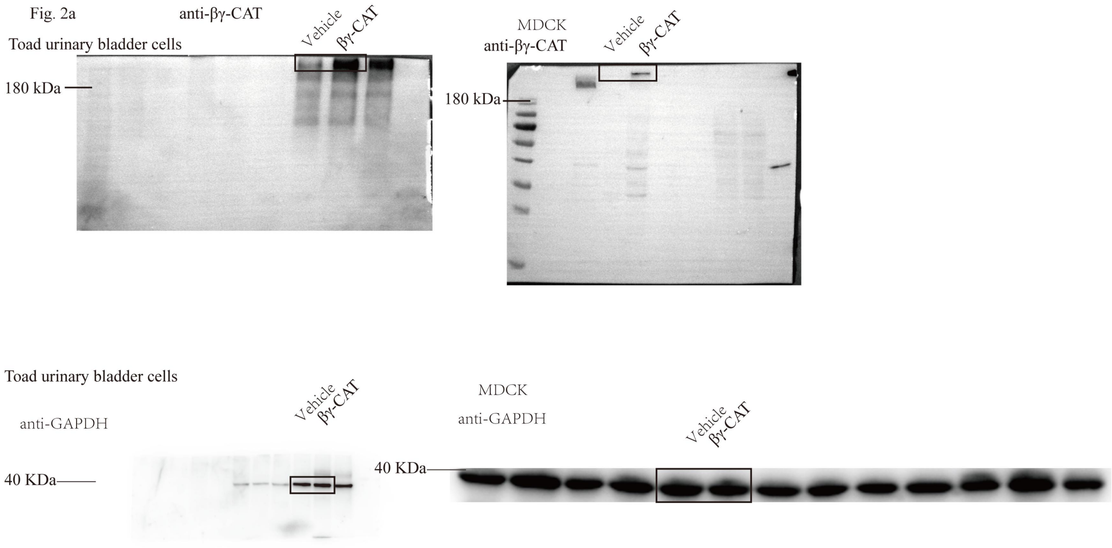

93

Fig. 3i MDCK

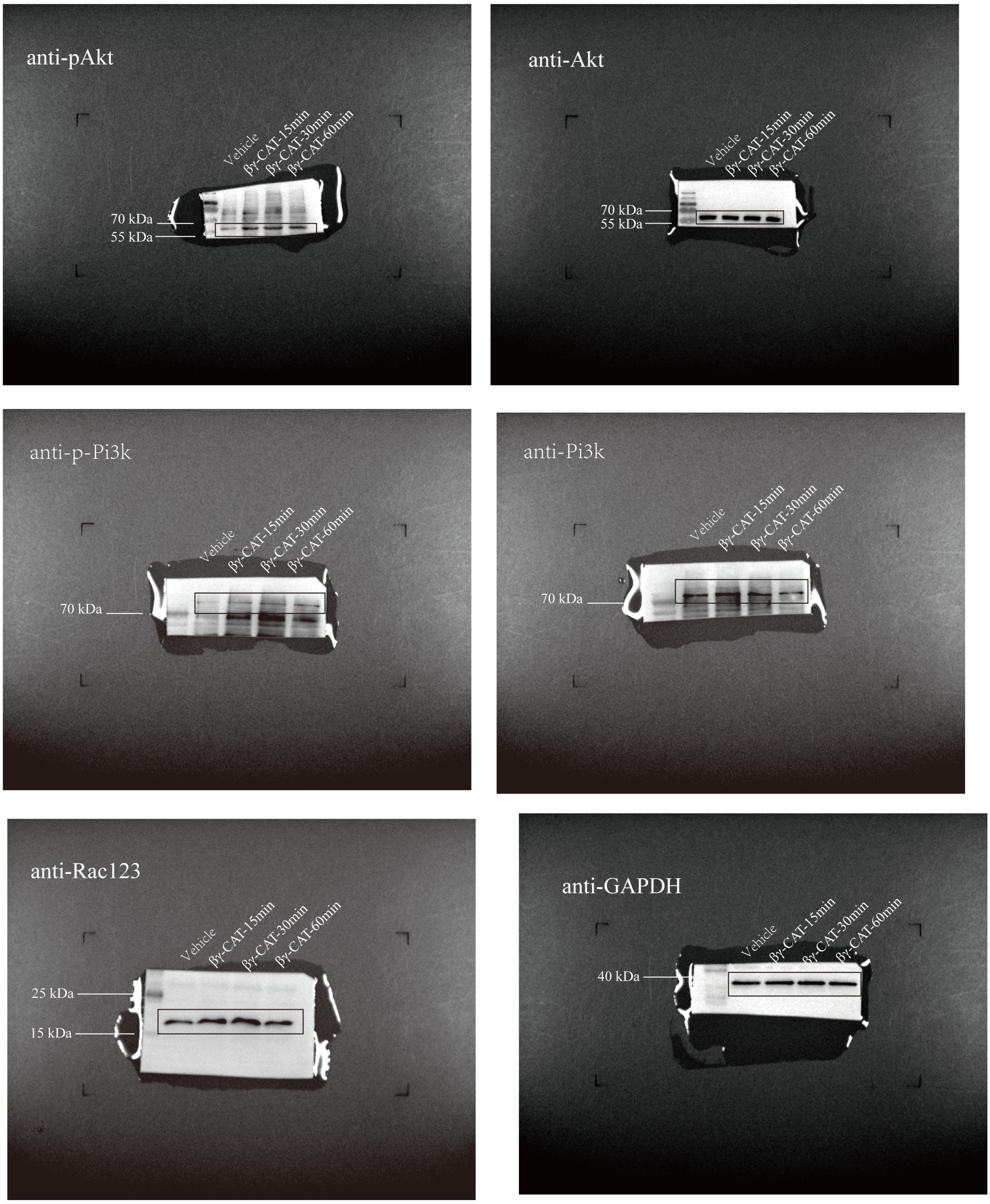

94

Fig.5c

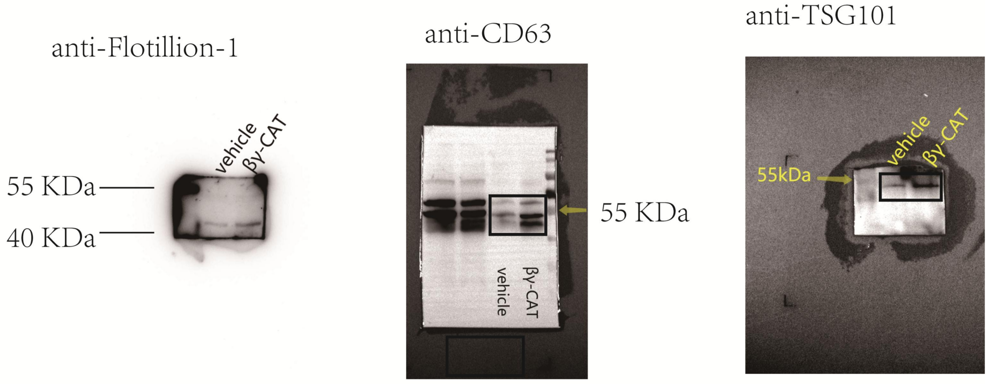

Fig.5h

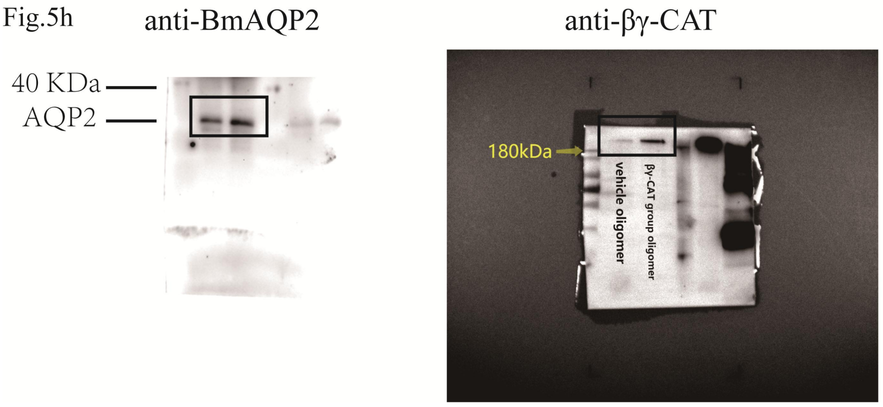

Fig.S2d

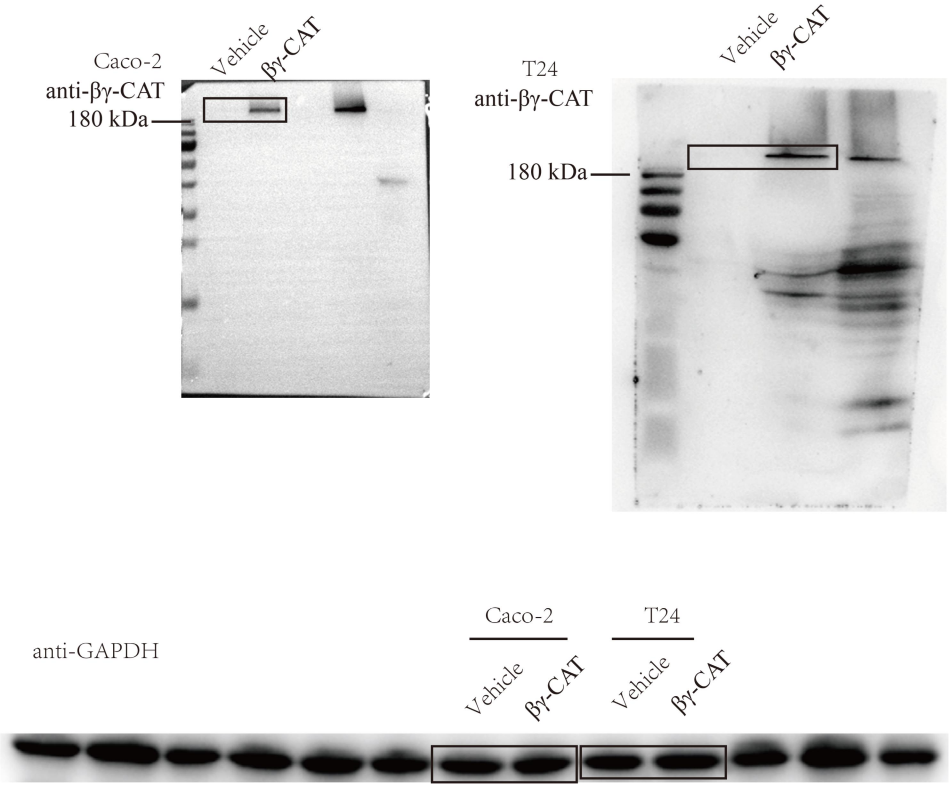

The area circled by the black square is the area shown in figures.

Supplementary Table 1. Sequences of primers used in this study

Name	Sequence
βγ-CAT-α-Forward	GCTTCCTCTCTGCGTGTGAT
βγ-CAT-α-Reverse	GCTTGATAACTGGGTCCCCC
βγ-CAT-β-Forward	GCAGCATATGACAGAATTGCATGTCC
βγ-CAT-β-Reverse	ACATCCAACTCTTTCTGCAGGGTC
β-actin-Forward	GTAGCCCCTGAAGAACACCC
β-actin-Reverse	TTGCATGGGGCAGAGCATAA
Bm-FcRn-Forward	GAGGCCGTATTCAAACACGAT
Bm-FcRn-Reverse	TTGGAGCCTTTGCATAACGA
