# Supplementary material for: A pore-forming protein drives macropinocytosis to facilitate toad water maintaining
Source: Commun Biol. 2022 Jul 22;5:730. doi: 10.1038/s42003-022-03686-1 (PMC9307623; doi:10.1038/s42003-022-03686-1)
Supplement: Supplementary file 3 — Reporting Summary [file 42003_2022_3686_MOESM3_ESM.pdf]

nature portfolio

Corresponding author(s): Yun Zhang

Last updated by author(s): Jul 8, 2022

Reporting Summary

Nature Portfolio wishes to improve the reproducibility of the work that we publish. This form provides structure for consistency and transparency in reporting. For further information on Nature Portfolio policies, see our Editorial Policies and the Editorial Policy Checklist.

Statistics

For all statistical analyses, confirm that the following items are present in the figure legend, table legend, main text, or Methods section.

n/a	Confirmed	
☐	☒	The exact sample size (n) for each experimental group/condition, given as a discrete number and unit of measurement
☐	☒	A statement on whether measurements were taken from distinct samples or whether the same sample was measured repeatedly
☐	☒	The statistical test(s) used AND whether they are one- or two-sided<br>*Only common tests should be described solely by name; describe more complex techniques in the Methods section.*
☐	☒	A description of all covariates tested
☐	☒	A description of any assumptions or corrections, such as tests of normality and adjustment for multiple comparisons
☐	☒	A full description of the statistical parameters including central tendency (e.g. means) or other basic estimates (e.g. regression coefficient) AND variation (e.g. standard deviation) or associated estimates of uncertainty (e.g. confidence intervals)
☐	☒	For null hypothesis testing, the test statistic (e.g. F, t, r) with confidence intervals, effect sizes, degrees of freedom and P value noted<br>*Give P values as exact values whenever suitable.*
☒	☐	For Bayesian analysis, information on the choice of priors and Markov chain Monte Carlo settings
☒	☐	For hierarchical and complex designs, identification of the appropriate level for tests and full reporting of outcomes
☒	☐	Estimates of effect sizes (e.g. Cohen's d, Pearson's r), indicating how they were calculated

Our web collection on statistics for biologists contains articles on many of the points above.

Software and code

Policy information about availability of computer code

Data collection	We state that no software was used in our study.
Data analysis	We state that no software was used in our study.

For manuscripts utilizing custom algorithms or software that are central to the research but not yet described in published literature, software must be made available to editors and reviewers. We strongly encourage code deposition in a community repository (e.g. GitHub). See the Nature Portfolio guidelines for submitting code & software for further information.

Data

Policy information about availability of data

All manuscripts must include a data availability statement. This statement should provide the following information, where applicable:

- Accession codes, unique identifiers, or web links for publicly available datasets
- A description of any restrictions on data availability
- For clinical datasets or third party data, please ensure that the statement adheres to our policy

Data availability: All data needed to evaluate the conclusions in the paper are present in the paper and/or the Supplementary Materials.
https://doi.org/10.5061/dryad.0p2ngf226

Field-specific reporting

Please select the one below that is the best fit for your research. If you are not sure, read the appropriate sections before making your selection.

☒ Life sciences ☐ Behavioural & social sciences ☐ Ecological, evolutionary & environmental sciences

For a reference copy of the document with all sections, see nature.com/documents/nr-reporting-summary-flat.pdf

Life sciences study design

All studies must disclose on these points even when the disclosure is negative.

Sample size	In our study, the size of samples (such as frogs, peritoneal cells) were determined based on our previous study. For example, the frogs used in our study are commonly weight of 19 ± 5 g , each group should be contain at least 5 frogs. For the size of cells, different number of cells were used in different assays, but we did at least duplicate in parallel.
Data exclusions	No data were excluded from the analyses
Replication	All of our assays are done at least three independent experiments and could be replicated successfully.
Randomization	In our study, the samples (such as frogs) were allocated into experimental groups completely random.
Blinding	Yes, we were blinded to group allocation during data collection and following analysis.

Reporting for specific materials, systems and methods

We require information from authors about some types of materials, experimental systems and methods used in many studies. Here, indicate whether each material, system or method listed is relevant to your study. If you are not sure if a list item applies to your research, read the appropriate section before selecting a response.

Materials & experimental systems

n/a	Involved in the study	
☐	☒	Antibodies
☐	☒	Eukaryotic cell lines
☒	☐	Palaeontology and archaeology
☐	☒	Animals and other organisms
☒	☐	Human research participants
☒	☐	Clinical data
☒	☐	Dual use research of concern

Methods

n/a	Involved in the study	
☒	☐	ChIP-seq
☐	☒	Flow cytometry
☒	☐	MRI-based neuroimaging

Antibodies

Antibodies used

The antibodies used in our study were listed as below:
1. βγ-CAT rabbit polyclonal antibody and mouse polyclonal antibody were prepared by our laboratory.
2.Anti-pan cytokeratin AE1/AE3 monoclonal antibody (Thermo Fisher Scientific, Rockford, IL, USA).
3.Mouse-derived anti-AQP2 (Santa Cruz, Cat sc-515798.
4.Rabbit-derived anti-AQP2 (ImmunoWay, Cat YT0290).
5.Rabbit-derived anti-PI3 Kinase p85 (CST, Cat 4257), rabbit-derived anti-phospho-PI3 Kinase p85 (CST, Cat 4228), rabbit-derived anti-Rac1/2/3 (CST, Cat 2465), rabbit-derived anti-total Akt (CST, Cat 4691) and rabbit-derived anti-phospho-Akt-S473 (CST, Cat 4060).
6.Rabbit-derived anti-TSG101 (Proteintech, Cat 28283-1-AP), Rabbit-derived anti-Flotillin 1 Polyclonal antibody (Proteintech, Cat 15571-1-AP)
7.Mouse-derived anti-CD63 (Abcam, Cat ab193349).
8.5-nm or 10-nm colloidal gold-conjugated secondary antibody (sigma, Cat G7527 and G7402).
9.HRP conjugated Goat anti-mouse IgG (H+L), Catalog number: SA00001-1, and HRP conjugated Goat anti-rabbit IgG (H+L), Catalog number: SA00001-2were purchased from Proteintech Group, Inc.
10.Alexa Fluor 488 conjugated Goat anti-Rabbit IgG (H+L) secondary antibody, Catalog number: SA00013-2, Alexa Fluor 488 conjugated Goat anti-Mouse IgG (H+L) secondary antibody, Catalog number: SA00013-1, and Alexa Fluor 594 conjugated Goat anti-Rabbit IgG (H+L) secondary antibody, Catalog number: SA00013-4, all were purchased from Proteintech Group, Inc.

Validation

All of the above primary antibodies were testified valid in our study. The detailed description as below:
1. βγ-CAT rabbit polyclonal antibody and mouse polyclonal antibody, species: Bombina maxima, application: WB, IEM and IF.
2.Anti-pan cytokeratin AE1/AE3 monoclonal antibody, species: Bombina maxima, application: FC.
3.Mouse-derived anti-AQP2 (Santa Cruz, Cat sc-515798),, species: Canine, Bombina maxima, application: IF.
4.Rabbit-derived anti-AQP2 (ImmunoWay, Cat YT0290), species: Bombina maxima, application: IF and WB.

5. Rabbit-derived anti-PI3 Kinase p85 (CST, Cat 4257), rabbit-derived anti-phospho-PI3 Kinase p85 (CST, Cat 4228), rabbit-derived anti-Rac1/2/3 (CST, Cat 2465), rabbit-derived anti-total Akt (CST, Cat 4691) and rabbit-derived anti-phospho-Akt-S473 (CST, Cat 4060), species: Canine, application: WB.
6.Rabbit-derived anti-TSG101 (Proteintech, Cat 28283-1-AP), Rabbit-derived anti-Flotillin 1 Polyclonal antibody (Proteintech, Cat 15571-1-AP), Mouse-derived anti-CD63 (Abcam, Cat ab193349), species: Bombina maxima, application: WB.

Eukaryotic cell lines

Policy information about cell lines

Cell line source(s)	MDCK cells (ATCC® CRL-2935™), Caco-2 (ATCC® HTB-37™), T24 (ATCC® HTB-4™)was purchased from ATCC.
Authentication	The MDCK, Caco-2 and T24 cells we used in our study were authenticated by Kunming Cell Bank of Type Culture Collection, CAS.
Mycoplasma contamination	The MDCK, Caco-2 and T24 cells we used in our study was tested negative for mycoplasma contamination.
Commonly misidentified lines (See ICLAC register)	There is no any misidentified cell line with MDCK, Caco-2 or T24 can be found in Database of Cross-Contaminated or Misidentified Cell Lines.

Animals and other organisms

Policy information about studies involving animals; ARRIVE guidelines recommended for reporting animal research

Laboratory animals	We state that our study did not involve laboratory animals.
Wild animals	We state that our study did not involve wild animals.
Field-collected samples	Toads (B. maxima) were captured in the wild and raised at room temperature by feeding with live Tenebrio molitor. Toads with an average weight of 19 ± 5 g were used in experiments after fasting in isotonic Ringer's solution for 3 days.
Ethics oversight	All the procedures and the care and handling of the animals were approved by the Institutional Animal Care and Use Committee at Kunming Institute of Zoology, Chinese Academy of Sciences (Approval ID: IACUC-OE-2021-05-001).

Note that full information on the approval of the study protocol must also be provided in the manuscript.

Flow Cytometry

Plots

Confirm that:

☒ The axis labels state the marker and fluorochrome used (e.g. CD4-FITC).

☒ The axis scales are clearly visible. Include numbers along axes only for bottom left plot of group (a 'group' is an analysis of identical markers).

☒ All plots are contour plots with outliers or pseudocolor plots.

☒ A numerical value for number of cells or percentage (with statistics) is provided.

Methodology

Sample preparation	2×105 MDCK and T24 cells were incubated with 100 µg/mL 70 kDa FITC-label dextran (Sigma, Cat 46945) or Lucifer Yellow (Sigma, Cat L0144) in the dark at 37? for 30 minutes with and without 10 nM or 5 nM βγ-CAT, respectively. The samples were followed by fluorescence detection of FITC or AmCyan. In each sample, 1×104 single cells were analyzed. 2×106 toad UB epithelial cells and peritoneal cells were treated with 50 nM βγ-CAT, while 100 nM βγ-CAT was used for 2×106 toad skin and kidney cells. In the test using immunodepletion of endogenous βγ-CAT, toad cells were incubated with 50 µg/mL rabbit-derived anti-βγ-CAT antibodies for 30 minutes before the above protocol was carried out. During the inhibitor experiment, cells were first incubated with 100 µM EIPA (MedChemExpress, Cat HY-101840A) or 20 µM wortmannin (Sigma, Cat 681675) for 1 hour at 37?. In addition, 2×106 digested toad UB epithelial cells were cultured in vitro for 3 hours and co-incubated with 500 ng/mL propidium iodide (Becton Dickinson, Franklin Lakes, NJ, USA) for 10 minutes at room temperature. Finally, the cells were resuspended with 300 µL PBS and analyzed with a flow cytometer.
Instrument	LSR Fortessa cell analyzer (Becton Dickinson, Franklin Lakes, NJ, USA)
Software	FlowJo 10
Cell population abundance	The abundance of cell population was mainly depended on the forward scatter (FSC) and side scatter (SSC) of MDCK, Caco-2, T24 cells or frog skin, UB, kidney and peritoneal cells. For MDCK, Caco-2 and T24 cells, the cell population was single and could be thought of as a group of pure cells.

Gating strategy

The gating we used was depended on the positive and negative control of our assay.

☐ Tick this box to confirm that a figure exemplifying the gating strategy is provided in the Supplementary Information.
